# Correlation between gold and mercury in human blood among individuals in northwestern Tanzania

**DOI:** 10.20935/acadphealth8321

**Published:** 2026-05-29

**Authors:** Zakir Bulmer, Megan Willkens, Robert N. Peck, Lily D. Yan, Elias C. Nyanza

**Affiliations:** 1 Center for Global Health, Department of Internal Medicine, Weill Cornell Medicine, New York, NY, USA; 2 Mwanza Intervention Trials Unit & National Institute for Medical Research (MITU/NIMR), Mwanza, Tanzania; 3 Department of Community Medicine, Environmental and Occupational Health, School of Public Health, Catholic University of Health and Allied Sciences, Mwanza, Tanzania

**Keywords:** Tanzania, Artisanal and small-scale gold mining, mercury, gold, exposure, biomarker

## Abstract

**Introduction:**

Mercury is a highly toxic metal of major global health concern, with exposure disproportionately affecting low- and middle-income countries (LMICs) where artisanal and small-scale gold mining (ASGM) is common. While mercury exposure has been well documented among mining populations, less is known about exposure in general urban populations.

**Materials and methods:**

We analyzed baseline data from 1000 adults enrolled in the Mwanza HIV and Cardiovascular Disease cohort (2015–2019) in northern Tanzania. Blood total mercury (T-Hg) and total gold (T-Au) concentrations were measured from dried blood spots using inductively coupled plasma mass spectrometry. Associations between T-Hg and T-Au concentrations were evaluated using Spearman’s rank correlation. Associations between *a priori*-selected covariates and T-Au concentrations were assessed using multivariable bootstrap linear regression models with 1000 resampling iterations.

**Results:**

All participants had detectable blood mercury levels, with a median T-Hg concentration of 5.02 μg/l, and 7.2% had levels exceeding the World Health Organization toxic threshold of ≥20 μg/l. Detectable T-Au was observed in approximately 60% of participants, with a median T-Au concentration of 0.09 μg/l. In bootstrap multivariable regression analyses, HIV infection was associated with higher T-Au concentrations (median β = 0.28; 95% bootstrap CI 0.12–0.51). T-Hg and T-Au concentrations were moderately positively correlated in Spearman’s rank correlation (ρ = 0.45; *p* < 0.001). After multivariable adjustment, T-Hg remained positively associated with T-Au concentrations (median β = 0.39; 95% bootstrap CI 0.22–0.58).

**Conclusions:**

We found that T-Hg and T-Au were detectable in a general population living near an ASGM site, and associated with each other, suggesting a common source of exposure. Improved characterization of exposure routes is critical for informing targeted interventions to reduce mercury-related health risks in Tanzanian communities.

## 1. Introduction

Mercury exposure affects 19 million people worldwide [1,2], with multiple adverse health outcomes including liver and kidney damage; gastric and vascular disorders; hormonal imbalances, miscarriages, and reproductive disorders; skin lesions; neurological disorders including visual impairment, ataxia, paresthesia, neurasthenia, hearing loss, dysarthria, neurodegeneration, and muscle tremor; and death [3,4]. Exposure to even low amounts can result in serious health effects, especially for developing fetuses and young children [5]. As such, the World Health Organization lists mercury among the ten most hazardous substances in the world [6].

The burden of mercury exposure disproportionately affects low- and middle-income countries (LMICs), like Tanzania, as common sources include mining, smelting, coal combustion, and waste burning [7]. Artisanal and small-scale gold mining (ASGM) is a major source of mercury exposure in Tanzania, as mercury has a natural affinity to gold and is used to extract gold from ore [8–11]. After mercury is added to crushed ore, the amalgam—or mixture of gold and mercury—is heated in an open-air environment to isolate purified gold, releasing toxic mercury vapors in the process [12,13]. Mining tailings—waste materials from the extraction process—are also discharged into the environment, polluting the surrounding soil and water. Containment strategies are often minimal or absent in most ASGM communities [14]. People are exposed to mercury when they consume mercury-contaminated fish, crops and soil (geophagy), breathe mercury vapor, and handle improperly purified gold products [15–21].

People engaged in ASGM activities have been shown to have significantly higher mercury levels as measured through multiple biomarkers. Gold miners in Mwakitolyo and Katente had significantly higher mercury levels in their urine compared to their non-mining counterparts [13]. Pregnant women in Northern Tanzania involved in mining activities, as well as those of lower socioeconomic status, had higher mercury levels in dried blood spots compared to women who were not involved in mining [18]. Residing in areas with ASGM activity has been associated with increased blood mercury levels [18]. A study of soil and food crops around Shenda gold mine in Geita, Tanzania, estimated weekly intake of mercury by measuring mercury levels in crops (e.g., rice) and average regional diets, calculating an estimated weekly mercury intake of 0.003 milligrams per kilogram of body weight per week (mg/kg bw/week) due to rice grain consumption, which is almost two times the provisional tolerable weekly intake of 0.0016 mg/kg bw/week set by the Joint Food and Agriculture Organization/World Health Organization (FAO/WHO) Expert Committee on Food Additives [22,23].

Despite this evidence, blood mercury levels among the general population who live near ASGM but do not engage in occupational ASGM, and risk factors for elevated blood mercury levels remain incompletely characterized. To fill this gap, we measured blood mercury and gold in a cohort of adults from Mwanza, Tanzania, who live near ASGM in Geita. We quantify blood mercury and gold levels, examine risk factors for elevated gold using multivariable analysis, and test whether the mercury–gold relationship remains significant after adjustment to determine if there may be a common source. Demonstrating an adjusted co-variation between these metals could strengthen inference about shared environmental or behavioral sources and help guide targeted biomonitoring and exposure-reduction priorities in ASGM-affected communities.

## 2. Materials and methods

### 2.1. Study design, setting, and population

We analyzed baseline, cross-sectional data from the Mwanza Human Immunodeficiency Virus (HIV) and Cardiovascular Disease cohort (2015–2019), a longitudinal study based in Mwanza, northern Tanzania. The full details of the cohort have been published elsewhere [24]. The cohort was originally established to examine links between HIV and cardiovascular outcomes among 1000 adults, comprising 495 people living with HIV (PLWH) and 505 HIV-negative “treatment partners” recruited from three public outpatient HIV clinics (Bugando Medical Centre, Igoma Health Centre, and Nyamagana District Hospital). Individuals with and without HIV had comparable socioeconomic profiles [25]. Mwanza, the country’s second largest city, located on the southern shore of Lake Victoria, has an economy dominated by fishing, agriculture, and artisanal and large-scale gold mining (ASGM), which provides roughly 800,000 jobs [11].

### 2.2. Data collection

We employed a validated, standardized questionnaire adapted from the World Health Organization’s STEPwise approach to noncommunicable disease (NCD) risk factor surveillance (STEPS) to gather data on participants’ sociodemographic and economic characteristics, such as occupation type—manual labor versus office-based work—exposure to tobacco smoke, alcohol consumption, dietary patterns (including primary drinking water source), and levels of physical activity [26]. We also measured height and weight to calculate body mass index (BMI) as an indicator of nutritional status and included additional questions on HIV diagnosis, care, and treatment. The WHO STEPS tool was validated and translated into Kiswahili—the primary language in the East African region [27]. The characteristics of the Mwanza HIV and Cardiovascular Disease (CVD) cohort have been published [24].

### 2.3. Mercury and gold blood levels

Mercury and gold were measured from biobanked dried blood spots (DBSs) collected at baseline using a locally validated protocol previously reported. Capillary blood obtained by a finger prick was deposited onto Whatman 903 filter paper and air-dried. DBS punches were digested by microwave-assisted closed-vessel acid digestion at 170 °C for 30 min, and analyses were performed by inductively coupled plasma mass spectrometry (ICP-MS, PerkinElmer, USA) at an ISO/IEC 17025-accredited laboratory (ALS Scandinavia, Sweden) [35]. Method detection limits were calculated from field blank filter papers (mean + 3 SD) to account for background contamination, following earlier work [18,28]. Toxic mercury was classified as ≥20 μg/l in accordance with the World Health Organization (WHO), the US Agency for Toxic Substances and Disease Registry (ATSDR), and German Environmental Survey IV [29,30].

### 2.4. Outcomes and statistical analysis

The primary outcomes—blood total mercury (T-Hg) and total gold (T-Au)—were treated as continuous measures. Associations between T-Hg and T-Au concentrations were assessed using Spearman’s rank correlation. For regression analyses, T-Au concentrations were analyzed in their original units, while T-Hg concentrations were log-transformed prior to inclusion as a predictor variable to reduce skewness. Data processing and statistical analyses were conducted in RStudio (version 2023.09.1).

### 2.5. Regression analysis

We fitted two multivariable linear regression models on the full sample (N = 1000): (1) a risk-factor model for T-Au that excluded T-Hg as a covariate, and (2) an adjustment model for T-Au that included T-Hg as a covariate to evaluate whether the Au-Hg association persisted after covariate adjustment. Candidate predictors were selected *a priori* from the literature and included: HIV status, age, sex, alcohol use in the past 12 months, primary water source for daily use, and occupation. Age and log-transformed T-Hg were treated as continuous variables; HIV status, sex, alcohol use (past 12 months—yes/no), water source (running house water vs. other) and occupation (manual vs. non-manual) were included as binary variables [31].

Because T-Au concentrations exhibited a highly skewed distribution, multivariable bootstrap linear regression was used for inference. For each model, 1000 bootstrap resampling iterations with replacement were performed. Median regression coefficients and 95% percentile bootstrap confidence intervals (2.5th and 97.5th percentiles) were calculated from the empirical distribution of the bootstrap estimates. Model diagnostics included assessment of multicollinearity and influential observations.

### 2.6. Ethical considerations

Ethical clearance for the study protocol was granted by three independent review bodies: the Catholic University of Health and Allied Sciences/Bugando Medical Centre Joint Ethics and Research Review Committee (CREC/635/2022), Weill Cornell Medicine (Protocol 1506016328), and the Tanzania National Institute for Medical Research (NIMR/HG/R.8c/Vol.1/1399). The entire study was performed in full compliance with Good Clinical Practice guidelines and included robust measures to safeguard participant well-being and minimize any potential risks. Prior to enrolment, every participant provided written informed consent. Involvement in the study remained completely voluntary and had no bearing on the standard of care or services they received at the health facility.

## 3. Results

### 3.1. Sociodemographic characteristics

Out of 1000 participants analyzed, the median age was 36, and 67.5% were female (Table 1). Most (63.3%) study participants had completed primary education and had a normal-weight BMI (55.2%). The majority (64.5%) of participants were also making less than 150,000 Tanzanian shillings per month, and just under half (49.5%) of the adult cohort included people living with HIV (PLWH).

### 3.2. Mercury and gold blood levels

Approximately 60% of the adult cohort showed detectable levels of T-Au in their blood at baseline (n = 599), with a median blood T-Au level of 0.09 μg/l (Table 2). For mercury, all participants had detectable T-Hg levels, with a median blood T-Hg level of 5.02 μg/l (Table 2). In total, 7.2% (n = 72) of participants had toxic levels (≥20 μg/l) of T-Hg in their blood.

### 3.3. Regression analysis

In multivariable bootstrap linear regression analyses using raw T-Au concentrations as the outcome variable (Table 3), Model 1 (excluding T-Hg) identified HIV (median β = 0.28; 95% bootstrap CI 0.12–0.51) as positively associated with T-Au concentration. In Model 2, log-transformed T-Hg was the strongest correlate of T-Au concentration (median β = 0.39; 95% bootstrap CI 0.22–0.58). HIV positivity remained positively associated with T-Au concentrations (median β = 0.19; 95% bootstrap CI 0.05–0.38), while office/desk occupation was associated with lower T-Au concentrations (median β = −0.24; 95% bootstrap CI −0.53 to −0.03).

## 4. Discussion

Among a general cohort of adults living in Mwanza, Tanzania, we found that detectable blood mercury was universal and that approximately 60% of participants had detectable blood gold concentrations. T-Hg and T-Au concentrations were moderately positively correlated in unadjusted analyses, and this relationship persisted after multivariable adjustment. In the fully adjusted multivariable bootstrap linear regression model (N = 1000), higher log-transformed T-Hg concentrations were associated with higher T-Au concentrations (median β = 0.39; 95% bootstrap CI 0.22–0.58). These findings suggest that mercury and gold exposures may arise from shared environmental or behavioral pathways in this population.

In the risk factor analysis (Model 1), HIV infection was associated with higher T-Au concentrations. In the fully adjusted model (Model 2), HIV infection remained positively associated with T-Au concentrations, and office-based occupation was associated with lower T-Au concentrations. The magnitude of these associations was smaller than that observed for T-Hg, suggesting mercury exposure may be a major correlate of blood gold concentrations in this cohort. Notably, HIV infection has also previously been identified as a predictor of mercury exposure, in addition to alcohol intake, obesity, and longer duration of indoor smoke exposure [31].

Prior studies have focused largely on measuring mercury in ASGM populations that live near mines or engage in mining activities. These largely cross-sectional analyses have revealed that residential proximity to ASGM activities, occupational participation in ASGM activities, and lower socioeconomic status increase risk of mercury exposure [13,18,23]. However, the full etiology of mercury exposure remains unclear, and other studies have identified multiple other health risk factors, including cosmetic usage, alcohol intake, obesity, indoor smoke exposure, HIV infection, older age, and female sex, among others [31,32]. Here we present evidence that mercury and gold share exposure pathways in Mwanza, Tanzania, by directly measuring levels of blood mercury and gold in 1000 adult participants.

We found that over 60% of this general population cohort have detectable gold blood levels. Gold is commonly found in the human body at very low concentrations, but since it is biologically inert and generally nontoxic, it poses little to no threat to human health [33]. Prior studies investigating mercury exposure have not measured gold levels in the blood; this is the first human biomonitoring study to examine blood gold levels and their association with mercury exposure.

We found a moderate positive correlation between blood mercury levels and gold exposure in adults living in Tanzania. We measured mercury and gold levels using dried blood spots, which is widely considered the most reliable method for mercury detection in the human body [34]. The results build upon prior studies, which link self-reported ASGM activities with blood mercury levels, but never directly measure blood gold as a proxy for ASGM activities. Our results suggest that Tanzanian adults with gold in their blood are also more likely to be exposed to mercury, either from contaminated crops, soil, water, air, or handling of insufficiently purified gold.

The strengths of this study include the use of a reliable and validated sampling method (dried blood spots analyzed via ICP-MS) in a large population (N = 1000). Limitations include the inability to determine the specific chemical species of gold and mercury (i.e., only total element concentrations were quantified), which may include the possibility of complexes formed between the two elements.

## 5. Conclusions

In summary, this study provides population-level evidence that both mercury and gold circulate broadly in adults living in Mwanza, and that their co-occurrence is non-random. The observed association between the two metals supports the hypothesis that mercury and gold share exposure pathways. These findings highlight the importance of investigating environmental and behavioral pathways through which gold and mercury enter the human body. Clarifying these exposure routes will be crucial for developing targeted interventions to mitigate mercury-related health risks in Tanzanian communities.

## Figures and Tables

**Table 1.** Sociodemographic characteristics of study participants (N = 1000).

Variable	Category	n	%
Age (years)	18–24	157	15.7
	25–34	303	30.3
	35–64	540	54
Sex	Female	675	67.5
	Male	325	32.5
Body mass index (BMI)	Underweight	152	15.2
	Normal weight	552	55.2
	Overweight	195	19.5
	Obese	99	9.9
Education level	No formal education	69	6.9
	Completed primary education	633	63.3
	At least secondary education	225	22.5
Monthly income (Tanzanian shillings)	Under 150,000 TZS	645	64.5
	150,000 to 1,000,000 TZS	343	34.3
	Over 1,000,000 TZS	11	1.1
Duration of home smoke exposure (years)	Under 10 years	313	31.3
	10 to 30 years	386	38.6
	30 to 60 years	101	10.1
Prevalent hypertension	No	944	94.4
	Yes	56	5.6
HIV status	No	505	50.5
	Yes	495	49.5

**Table 2.** Mean and median concentrations of gold and mercury measured via ICP-MS analysis of dried blood spots from 1000 adults in northern Tanzania.

Metal	Mean (SD), μg/l	Median (1st quartile and 3rd quartile), μg/l
T-Au	0.39 (1.62)	0.09 (0.0, 0.30)
T-Hg	7.73 (8.33)	5.02 (3.11, 9.55)

Spearman’s rank correlation showed a moderate positive monotonic association between T-Hg and T-Au concentrations in original units (ρ = 0.45, *p* < 2.0 × 10^−16^).

**Table 3.** Correlates of blood gold concentration in multivariable bootstrap linear regression models using raw T-Au concentrations as the outcome variable: Model 1 (excluding T-Hg) and Model 2 (including log-transformed T-Hg). Median β coefficients and 95% percentile bootstrap confidence intervals were derived from 1000 bootstrap iterations (N = 1000).

Variable	Median β (95% bootstrap CI)	
	Model 1	Model 2
HIV status		
Positive	0.28 (0.12, 0.51)	0.19 (0.05, 0.38)
Negative	*Ref*	*Ref*
Age	0.00 (−0.01, 0.01)	0.00 (−0.01, 0.01)
Sex		
Female	0.14 (−0.06, 0.36)	0.15 (−0.06, 0.37)
Male	*Ref*	*Ref*
Alcohol in past year?		
No	−0.07 (−0.24, 0.14)	0.00 (−0.15, 0.20)
Yes	*Ref*	*Ref*
Water source		
Other	−0.17 (−0.41, 0.00)	−0.17 (−0.41, 0.01)
Running water in house	*Ref*	*Ref*
Occupation		
Office work	−0.20 (−0.49, 0.01)	−0.24 (−0.53, −0.03)
Manual work	*Ref*	*Ref*
Log-transformed T-Hg		0.39 (0.22, 0.58)

Ref = Reference category, T-Au = blood total gold, T-Hg = blood total mercury

## Data Availability

The data supporting the findings of this publication cannot be made available due to legal concerns regarding the Health Insurance Portability and Accountability Act (HIPAA).
